# A retrospective cohort and machine learning study on the efficacy, safety, and recurrence prediction of combined EI-VOM and linear ablation in persistent Non-valvular atrial fibrillation

**DOI:** 10.3389/fcvm.2026.1689942

**Published:** 2026-02-02

**Authors:** Ting Zhang, Yuling Dai, Wenzhai Cao

**Affiliations:** 1 Department of Health Humanities Research Center, Sichuan Vocational College of Health and Rehabilitation, Zigong, Sichuan, China; 2 Department of Cardiology, Jinniu District People’s Hospital, Chengdu, Sichuan, China; 3 Department of Cardiology, First People’s Hospital of Zigong City, Zigong, Sichuan, China

**Keywords:** atrial fibrillation, catheter ablation, machine learning, risk prediction, vein of Marshall

## Abstract

**Purpose:**

Although circumferential pulmonary vein isolation (CPVI) is the standard treatment for persistent nonvalvular atrial fibrillation (PeNVAF), its efficacy remains limited. Combining CPVI with linear ablation and ethanol infusion into the vein of Marshall (EI-VOM) (CPVI + PLUS) may improve patient outcomes.

**Method:**

This single-center retrospective study included hospitalized patients with PeNVAF who underwent their first radiofrequency ablation at Zigong First People's Hospital from August 2022 to August 2024. After propensity score matching (PSM), 132 patients were assigned to each group: the CPVI + PLUS group (combined EI-VOM and linear ablation) and the CPVI-only group. Outcomes were assessed at 3 and 6 months, including AF recurrence, complications, and cardiac function. Quality of life was evaluated at 6 months using the Atrial Fibrillation Effect on Quality-of-Life Questionnaire (AFEQT). The primary endpoints were AF recurrence rate and arrhythmia-free survival at 6 months after ablation; secondary endpoints included changes in cardiac function parameters, quality-of-life scores, and complication rates. Kaplan–Meier analysis was used to compare arrhythmia-free survival between the two groups, and univariate and multivariate logistic regression analyses were performed to identify risk factors for AF recurrence. In addition, a stacking ensemble model was developed to enhance prediction of AF recurrence after ablation.

**Results:**

There were no significant differences in baseline demographics, comorbidities, laboratory, or echocardiographic parameters between the two groups. The CPVI + PLUS group had a longer procedure time than the CPVI group, but intraoperative complication rates were similar. At 6-month follow-up, the CPVI + PLUS group showed significantly better AFEQT, left ventricular ejection fraction (LVEF), left atrial diameter(LAD), and left ventricular end-diastolic diameter (LVEDD) compared to the CPVI group. Recurrence rates of AF were lower in the CPVI + PLUS group, and arrhythmia-free survival time was longer. Multivariate analysis identified coronary artery disease, heart failure, severe preoperative valvular regurgitation, intraoperative myocardial injury, longer AF course, and larger LAD as independent risk factors for recurrence. Additional ablation strategies and left atrial appendage occlusion were protective factors. The stacking ensemble model achieved high accuracy, precision, recall, F1 score, and ROC-AUC in both training and validation cohorts. Feature importance analysis consistently identified AF course, age, body mass index, B-type natriuretic peptide, LVEF, platelet count, and uric acid as core predictors of recurrence risk.

**Conclusion:**

CPVI + PLUS significantly improved arrhythmia-free survival, cardiac function, and quality of life in patients with PeNVAF without increasing procedural risk. Multivariate and machine learning analyses confirmed that additional ablation and left atrial appendage occlusion were protective against recurrence, while heart failure, valvular regurgitation, and longer AF course increased risk. The stacking model showed excellent predictive performance, and key features identified may support precise risk stratification and individualized therapy.

## Introduction

Atrial fibrillation (AF) is a chronic and progressive arrhythmia, with over half of patients with paroxysmal AF eventually developing persistent AF (PeAF), significantly increasing the risk of heart failure and stroke (1). With an aging population, the societal and healthcare burden of AF continues to rise. Large-scale epidemiological surveys in China from 2014 to 2016 reported an AF prevalence of 1.8% among individuals aged 45 and above, with rates increasing sharply with age (2). Based on these data and the 2020 national census, the estimated number of AF patients in China is around 12 million (2). However, the true prevalence is likely higher, as about one-third of cases, especially paroxysmal AF, remain undiagnosed.

Catheter ablation has become a first-line treatment for AF, with the potential to intervene in the disease process and delay progression to PeAF (3). However, in patients with PeAF, the efficacy of circumferential pulmonary vein isolation (CPVI) alone is relatively limited (4). The most commonly adopted ablation strategy is CPVI combined with linear ablation, a technique derived from the surgical maze procedure using bipolar ablation (5), which aims to anatomically isolate the atria and interrupt the mechanisms sustaining AF. A large randomized controlled trial by Verma et al. (6) showed that adding linear ablation or complex fractionated atrial electrogram ablation to CPVI did not significantly improve outcomes in patients with PeAF. Recent studies have identified the vein of Marshall (VOM) as an emerging ablation target that is receiving increasing attention. The VOM is an embryological remnant of the left superior vena cava and is present in approximately 95% of patients. Together with the Marshall bundle, it is located within the epicardial fold known as the ligament of Marshall-a major barrier to achieving complete bidirectional block (7, 8). The VOM is adjacent to the mitral isthmus, rich in sympathetic and parasympathetic fibers, and electrically connected to the atrial myocardium. Valderrabano et al. (9) first introduced ethanol infusion into the vein of Marshall (EI-VOM) as a novel strategy for AF ablation. Given the unique anatomical and electrophysiological characteristics of the VOM, Schurmann et al. (10) found that ethanol rapidly penetrates cell membranes, disrupts their structure, and alters protein conformation, leading to rapid cell apoptosis or necrosis. When ethanol is infused via the VOM into the local myocardium, it exerts a potent cytotoxic effect, forming scar tissue and achieving ablation. A prospective, multicenter randomized controlled trialsystematically evaluated the efficacy of EI-VOM combined with catheter ablation vs. catheter ablation alone in patients with PeAF (11). The results showed that the combined EI-VOM group had a significantly higher 12-month arrhythmia-free survival rate compared to the CPVI-only group, as well as a lower rate of repeat ablation. This study provided the first evidence that EI-VOM can effectively improve ablation success rates. However, due to some heterogeneity in ablation strategies between groups, the independent effect of VOM ablation still requires further validation.

Moreover, there is a lack of systematic evaluation regarding the efficacy and safety of EI-VOM, and comparative studies between EI-VOM and conventional catheter ablation strategies remain limited. Therefore, this study aims to retrospectively analyze the efficacy and safety of the CPVI + PLUS ablation strategy in the treatment of PeAF. Additionally, we seek to develop a predictive model for AF recurrence using machine learning (ML) algorithms and to identify key clinical factors influencing ablation outcomes, with the goal of providing scientific evidence for individualized treatment strategies.

## Methods

### Patient population and study design

**Figure 1:**
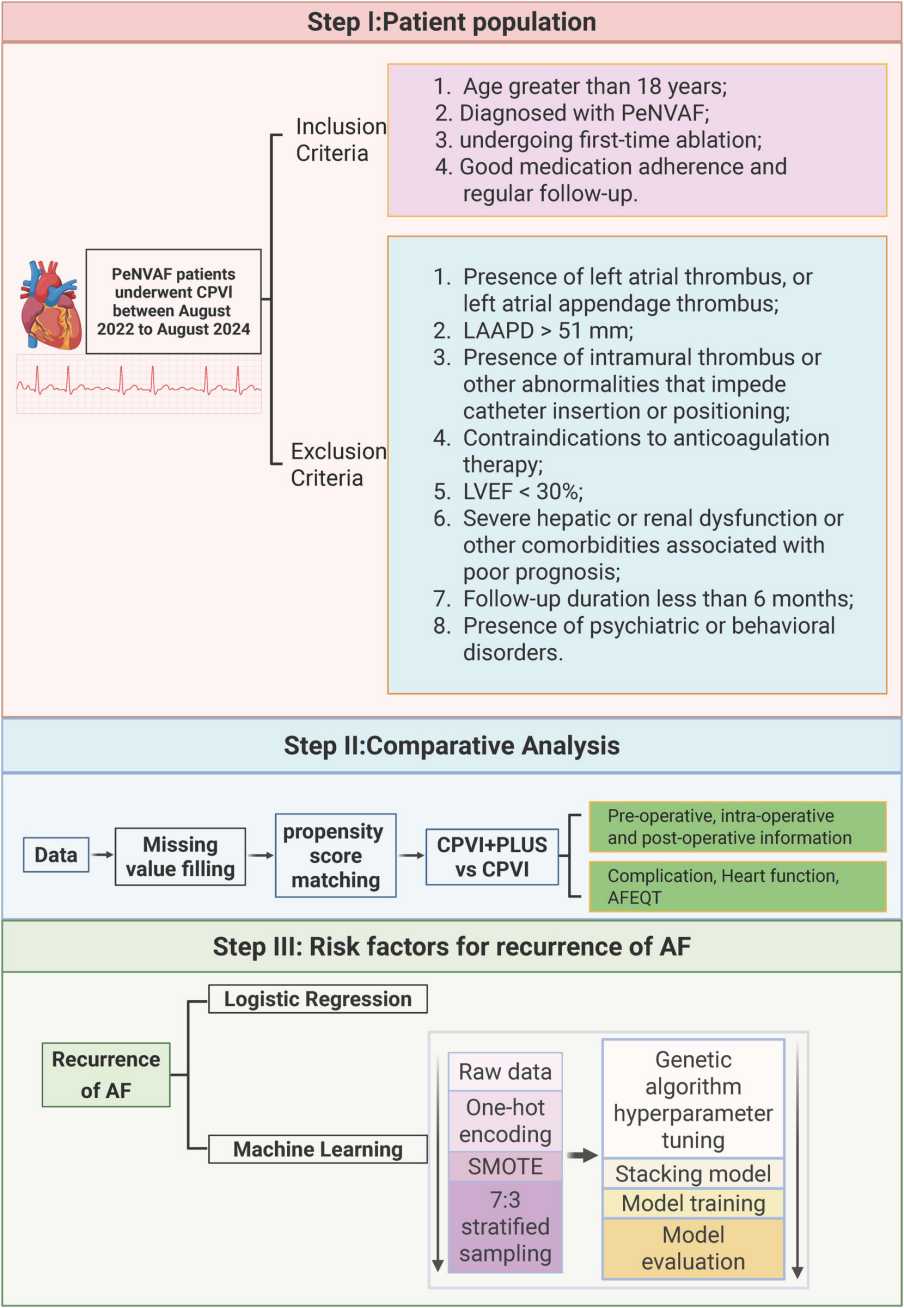
Study design flow chart. Created in BioRender. Xiong, T. (2026) licensed under Academic License.

This retrospective, single-center observational study enrolled consecutive patients hospitalized in the Department of Cardiology at Zigong First People's Hospital between August 2022 and August 2024 with a confirmed diagnosis of persistent atrial fibrillation (PeAF) who underwent first-time radiofrequency catheter ablation. All patients met catheter ablation indications according to the Chinese Guidelines for the Diagnosis and Management of Atrial Fibrillation (12). The study adopted a broad informed-consent framework and was approved by the Ethics Committee of Zigong First People's Hospital (approval No. 2025-037). The overall study workflow is shown in Figure 1.

### Eligibility criteria

Inclusion criteria were: (1) age ≥18 years; (2) diagnosis of PeAF and undergoing first-time ablation; (3) nonvalvular AF (NVAF); and (4) good medication adherence and regular follow-up.

Exclusion criteria were: (1) left atrial (LA) or left atrial appendage (LAA) thrombus on preoperative CT or transesophageal echocardiography (TEE); (2) LA anteroposterior diameter (LAAPD) > 51 mm; (3) intramural thrombus or other anatomic conditions interfering with catheter insertion/positioning; (4) contraindication to anticoagulation; (5) left ventricular ejection fraction (LVEF) <30%; (6) severe hepatic/renal dysfunction or other comorbidities associated with poor prognosis; (7) follow-up <6 months; or (8) psychiatric/behavioral disorders interfering with follow-up.

### Definitions and outcome ascertainment

#### Clinical definitions

PeAF was defined as AF that does not terminate spontaneously, with many interventional studies using ≥7 days to distinguish PeAF. Long-standing PeAF was defined as continuous AF lasting ≥12 months, which differs from permanent AF in that rhythm control may still be pursued. NVAF was defined as AF in the absence of rheumatic mitral stenosis, mechanical/bioprosthetic valve replacement, or prior mitral valve repair.

### Procedural effectiveness (acute endpoints)

Acute procedural effectiveness was defined by achievement of prespecified electrophysiological endpoints, including:1. confirmed complete electrical isolation of all pulmonary veins; and 2. when linear lesions were performed, confirmation of bidirectional block across the target lines (roof line, mitral isthmus, and/or floor line), as applicable.

### Arrhythmia recurrence (primary effectiveness endpoint)

Post-ablation recurrence was defined as documentation of AF/atrial flutter/atrial tachycardia lasting >30 s on a 12-lead electrocardiogram (ECG) or 24-hour Holter monitoring after a 3-month blanking period. Arrhythmias occurring within the first 3 months were classified as early recurrence and were not counted as the primary recurrence endpoint because a substantial proportion may resolve spontaneously.

### Safety endpoints

Acute complications included pericardial tamponade, cardiac perforation, severe bleeding, infection, and other adverse events occurring during the procedure or early recovery. Long-term complications included thromboembolism, worsening heart failure, and pulmonary vein stenosis.

### Periprocedural workflow and ablation strategy

#### Preoperative evaluation

All patients underwent TEE to exclude LA/LAA thrombus prior to ablation. Pulmonary vein CT angiography was performed to assess pulmonary vein anatomy and detect vascular anomalies. For patients unable to tolerate TEE, intracardiac echocardiography (ICE) was used intraoperatively to exclude thrombus before left atrial access.

#### CPVI + PLUS strategy

Under local or general anesthesia, femoral venous access was obtained using the Seldinger technique. A coronary sinus catheter was positioned to mark the mitral annulus. Transseptal puncture was performed via a modified Brockenbrough technique, and an 8.5F sheath (SL1, St Jude Medical, USA) was advanced into the left atrium. Intravenous heparin was administered to maintain an activated clotting time of 300–350 s.

For EI-VOM, a 6F JR4.0 guiding catheter was introduced into the coronary sinus for venography to identify the vein of Marshall (VOM). When visualized, a BMW guidewire (Abbott, USA) was advanced to the distal VOM, followed by placement of an 8 mm × 2 mm OTW balloon (Boston Scientific, USA) inflated to 6–8 atm. Ethanol (95%) was infused in 2-mL aliquots, each maintained for 120 s, with balloon repositioning to mid/proximal VOM as needed (total 4–10 mL). During infusion, bipolar potentials were monitored, and ethanol infusion was stopped once isthmus potentials were reduced by >90%.

#### Radiofrequency ablation (both groups)

Using CARTO 3 (Biosense Webster, USA), a high-density mapping catheter (Pentaray) was used to reconstruct left atrial anatomy and generate a voltage map. A contact force–sensing ablation catheter was used to perform CPVI, with confirmation of complete entrance/exit block. In the CPVI + PLUS group, additional lesions (roof line, mitral isthmus line, and/or left atrial floor line) were performed according to intraoperative findings, with verification of bidirectional block. Supplemental coronary sinus or other site ablation was performed when indicated. If typical atrial flutter was present pre- or intraoperatively, cavotricuspid isthmus ablation was performed. If patients remained in AF/atrial flutter after the lesion set, pharmacological cardioversion or direct current cardioversion (150–200 J) was performed to restore sinus rhythm.

#### Postoperative management and follow-up

All patients received antiarrhythmic drugs and anticoagulation for 3 months post-ablation. Follow-up was performed at 3 and 6 months. Recurrence assessment relied on scheduled ECG and/or 24-h Holter at follow-up visits, supplemented by additional ECG evaluation when symptoms occurred. At 6 months, quality of life was assessed using the Atrial Fibrillation Effect on Quality-of-Life (AFEQT) questionnaire, and echocardiography was performed to evaluate cardiac structure and function (including LAAPD and LVEF) (13).

### Data preprocessing and propensity score matching

#### Missing data

Missing values were handled using multiple imputation prior to downstream analyses. The imputation model incorporated baseline demographic, clinical, laboratory, and echocardiographic variables used in subsequent analyses to reduce bias associated with incomplete data.

#### PSM framework

To mitigate baseline imbalance between treatment strategies, propensity score matching (PSM) was performed using the following covariates: age, sex, body mass index (BMI), hypertension, diabetes, coronary artery disease, heart failure, prior stroke, valvular heart disease, LAAPD, left ventricular end-diastolic diameter (LVEDD), LVEF, CHA₂DS₂-VASc score, HAS-BLED score, high-sensitivity cardiac troponin T(hs-cTnT), B-type natriuretic peptide (BNP), albumin, and lipoprotein(a). After matching, patients were assigned to the CPVI + PLUS group (*n* = 132) or CPVI-only group (*n* = 132) based on the actual ablation strategy.

To enhance interpretability, baseline covariate balance was evaluated after matching (e.g., by standardized mean differences and/or balance plots), and the matched cohort was used as the primary analytic sample for group comparisons and outcome analyses.

### Machine-Learning model development and evaluation

#### Outcome, features, and data split

AF recurrence was defined as the outcome label, and all remaining variables served as candidate features. The dataset was split into training and validation sets at a 7:3 ratio using stratified sampling to preserve class proportions.

#### Class imbalance handling

To address class imbalance, Synthetic Minority Over-sampling Technique (SMOTE) was applied within the model-development pipeline (with care taken to avoid information leakage from validation data).

### Model construction and hyperparameter optimization

A stacking ensemble was developed with random forest (RF), extreme gradient boosting (XGBoost), logistic regression (LR), and support vector machine (SVM) as base learners and LR as the meta-classifier. Hyperparameters were optimized using a genetic algorithm implemented in the distributed evolutionary algorithms in python (DEAP) framework. Each individual encoded six hyperparameters: number of trees (RF, XGBoost), maximum depth (XGBoost), regularization parameter C (LR, SVM), and gamma (SVM). In each generation, mean area under the curve (AUC) from five-fold stratified cross-validation (*n* = 5) served as the fitness score. The genetic algorithm was run for 10 generations with a population size of 20, using crossover and Gaussian mutation. The hyperparameter set with the best cross-validated AUC was selected, and the final stacking model was trained on the training set.

### Model performance metrics

Model performance was evaluated on both training and validation sets using accuracy, precision, recall, F1 score, and area under the receiver operating characteristic curve (ROC-AUC). To enhance transparency, model diagnostics (e.g., confusion matrix and decision curve analysis) were reported.

### Statistical analysis

Analyses were performed in R 4.3.0, and ML modeling was performed in Python 3.12.5. Continuous variables were assessed for normality and compared using Student's *t*-test or the Mann–Whitney *U*-test, as appropriate; multi-group comparisons used analysis of variance (ANOVA) or Kruskal–Wallis tests. Categorical variables were compared using *χ*^2^ or Fisher's exact tests. AF-free survival was analyzed using Kaplan–Meier curves and compared with the log-rank test. Univariate logistic regression identified candidate predictors of recurrence; statistically significant variables were entered into multivariable logistic regression to determine independent associations.

## Results

### Comparison of preoperative baseline between CPVI and CPVI + PLUS

In this study, there were no statistically significant differences (*P* > 0.05) between the CPVI and CPVI + PLUS groups in terms of demographic characteristics, clinical features, or major laboratory and echocardiographic parameters (Supplementary Table S1). Specifically, age, sex, height, weight, BMI, AF course, CHA₂DS₂-VASc score, HAS-BLED score, smoking and drinking history, major comorbidities (such as hypertension, diabetes, coronary artery disease, previous stroke, and heart failure), laboratory indices (including creatinine, uric acid, albumin, fasting blood glucose (FBG), total cholesterol (TC), triglycerides (TG), high/low-density lipoprotein cholesterol (H/LDL-C), lipoprotein(a), hs-cTnT, platelet count (PLT), and BNP, and echocardiographic measurements (LVEF, LAAPD, LVEED, and the incidence of severe valvular regurgitation) were all comparable between the two groups. The good comparability of baseline characteristics provides a solid foundation for subsequent analyses of efficacy and prognosis.

### Comparison of intraoperative parameters between CPVI and CPVI + PLUS

There was no significant difference in the proportion of left atrial appendage occlusion between the CPVI and CPVI + PLUS groups (40.9% vs. 48.5%, *P* = 0.265). The CPVI + PLUS group had significantly higher rates of additional linear ablation (mitral isthmus line, left atrial top line, left atrial bottom line, and tricuspid isthmus line; all *P* < 0.001), whereas none of the patients in the CPVI group underwent these procedures. Cryoablation was predominantly used in the CPVI group (88.6%), while all patients in the CPVI + PLUS group received radiofrequency ablation. There was no significant difference in sinus rhythm restoration methods or mean number of electrical cardioversions between groups (*P* = 0.120 and *P* = 0.792, respectively). However, the mean procedure time was significantly longer in the CPVI + PLUS group (205.8 ± 59.6 min vs. 151.8 ± 61.5 min, *P* < 0.001), indicating increased procedural complexity in CPVI + PLUS groups (Supplementary Table S2).

### Comparison of intraoperative and postoperative complications between CPVI and CPVI + PLUS

There were no statistically significant differences in the incidence of myocardial injury (6.8% vs. 3.0%, *P* = 0.255), sinus bradycardia (2.3% vs. 1.5%, *P* = 1.000), pericardial effusion (2.3% vs. 3.0%, *P* = 1.000), or puncture site complications (2.3% vs. 1.5%, *P* = 1.000) between the CPVI and CPVI + PLUS groups. Despite the increased procedural complexity in the CPVI + PLUS group, no significant increase in perioperative complication risk was observed, indicating good surgical safety and clinical feasibility (Supplementary Table S3).

### Comparison of postoperative follow-up outcomes between CPVI and CPVI + PLUS

At 6-month follow-up (Supplementary Table S4), the CPVI + PLUS group demonstrated significantly better quality of life scores than the CPVI group (72.2 ± 7.1 vs. 66.4 ± 7.9, *P* = 0.000). Postoperative LVEF was higher (62.6 ± 6.3% vs. 59.9 ± 6.3%, *P* = 0.000), and both LAAPD and LVEDD were lower (37.0 ± 4.8 mm vs. 38.8 ± 7.7 mm, *P* = 0.003; 45.2 ± 7.8 mm vs. 47.8 ± 4.4 mm, *P* = 0.006) in the CPVI + PLUS group, indicating improved cardiac function. There were no significant differences between groups in the incidence of severe valvular regurgitation (38.6% vs. 41.7%, *P* = 0.706) or heart failure (50.0% vs. 53.8%, *P* = 0.622) postoperatively. The AF recurrence rate was significantly lower in the CPVI + PLUS group (10.6% vs. 27.3%, *P* = 0.001), and AF-free survival was longer (5.8 ± 0.7 vs. 4.9 ± 1.8 months, *P* = 0.000).

**Figure 2:**
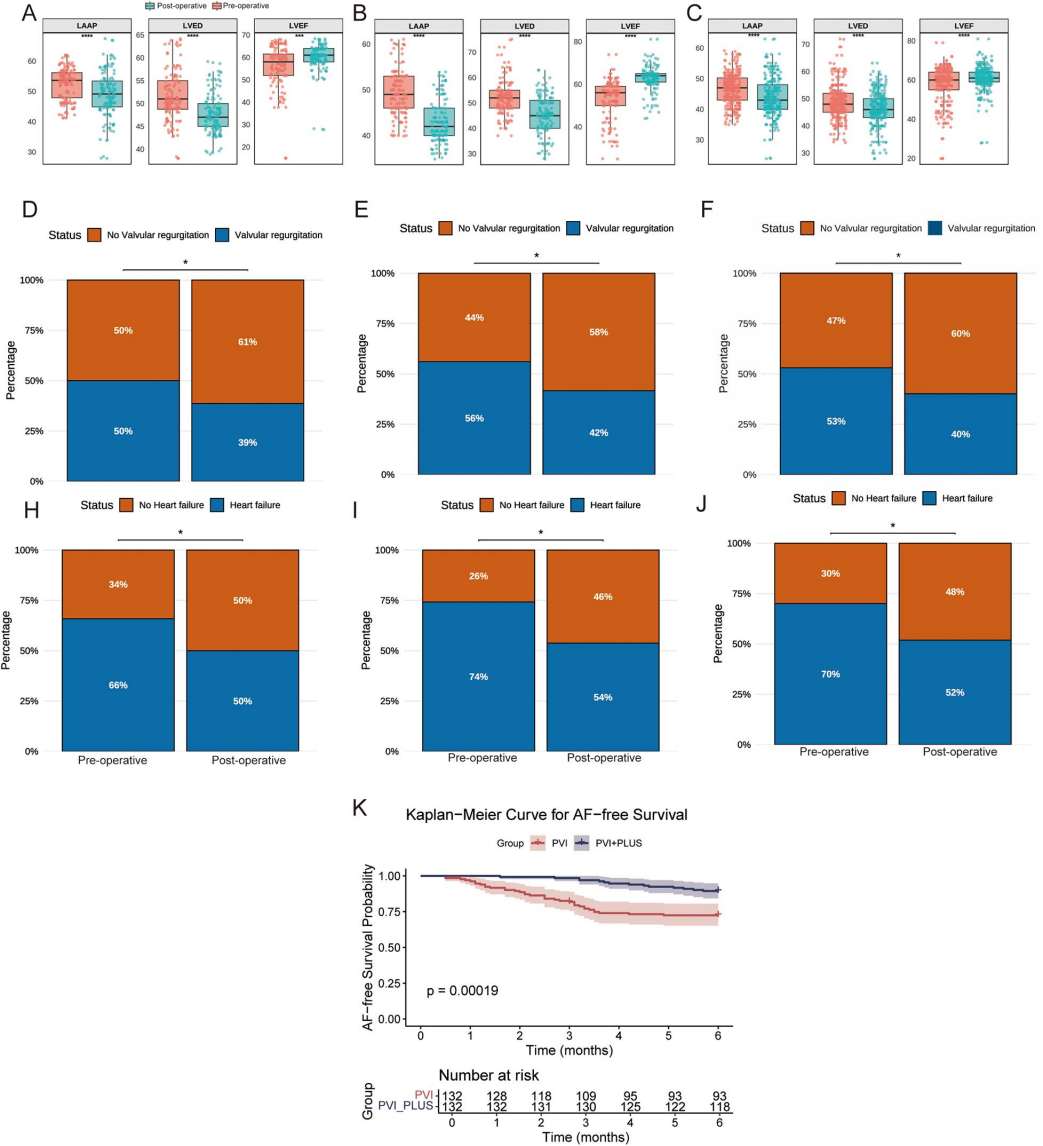
Clinical comparison of different groups before and after surgery. **(A–C)** The CPVI group, CPVI + PLUS group, and the overall cohort (ALL), respectively, showing pre- and post-operative changes in left atrial anteroposterior diameter (LAAP), left ventricular end-diastolic diameter (LVEDD), and left ventricular ejection fraction (LVEF). **(D–F)** The CPVI group, CPVI + PLUS group, and the overall cohort (ALL), respectively, show the incidence of valvular regurgitation before and after ablation; **(H–J)** The CPVI group, CPVI + PLUS group, and the overall cohort (ALL), respectively, show the incidence of heart failure. **(K)** displays the Kaplan–Meier curves for AF-free survival in the CPVI and CPVI + PLUS groups.

Both the CPVI and CPVI + PLUS groups, as well as the overall cohort, showed postoperative decreases in LAAPD and LVEDD, and increases in LVEF (Figures 2A–C). Additionally, both groups and the overall cohort showed significant reductions in the incidence of heart failure and valvular regurgitation compared to preoperative rates (*P* < 0.05, Figures 2D–J).

### AF-Free survival curves for the CPVI and CPVI + PLUS groups

At 6-month follow-up, the AF-free survival rate remained consistently higher in the CPVI + PLUS group compared to the CPVI group, with the gap between groups widening over time. By month 6, the recurrence-free survival probability was significantly lower in the CPVI group, while the CPVI + PLUS group maintained a higher rate. Log-rank analysis showed a highly significant difference between the two groups (*P* = 0.00019), indicating that the CPVI + PLUS strategy was more effective in reducing AF recurrence (Figure 2K).

### LR analysis of risk factors for AF recurrence

Among 264 patients, 50 experienced AF recurrence and 214 did not. Next, we compared the clinical characteristics before, during and after surgery between the group with postoperative AF recurrence and the group without postoperative AF recurrence (Supplementary Table S5). The recurrence group had a significantly higher proportion of patients receiving CPVI alone (72.0% vs. 44.9%, *P* = 0.001), while fewer underwent the combined CPVI + PLUS strategy, suggesting that combined ablation may reduce recurrence risk. Although age, sex, and BMI did not differ significantly between groups, the recurrence group had higher CHA_2_DS_2_-VASc scores and longer AF course (*P* < 0.05), indicating a greater burden of underlying disease. Coronary artery disease and heart failure were also more prevalent in the recurrence group (*P* = 0.012 and *P* = 0.027). Preoperative LAAPD and severe valvular regurgitation were significantly higher in the recurrence group (*P* = 0.002 and *P* < 0.001), reflecting more severe atrial remodeling and valvular dysfunction. Intraoperatively, the recurrence group had higher rates of cryoablation but lower rates of radiofrequency ablation, left atrial appendage occlusion, and mitral isthmus ablation. Myocardial injury was also more frequent in this group (*P* = 0.020). Postoperative assessments showed larger LAAPD and higher rates of severe valvular regurgitation and heart failure (all *P* < 0.05).

Univariate LR identified several variables significantly associated with postoperative AF recurrence (Supplementary Table S6). Additional ablation strategies (OR = 0.316, 95% CI: 0.157–0.608, *P* = 0.001), mitral isthmus ablation (OR = 0.387, 95% CI: 0.175–0.787, *P* = 0.013), and left atrial appendage occlusion (OR = 0.322, 95% CI: 0.154–0.633, *P* = 0.002) were associated with a reduced risk of recurrence, indicating potential protective effects. In contrast, coronary artery disease (OR = 2.311, *P* = 0.009), heart failure (OR = 2.607, *P* = 0.020), preoperative severe valvular regurgitation (OR = 4.560, *P* < 0.001), cryoablation (OR = 3.004, *P* = 0.001), and intraoperative myocardial injury (OR = 4.032, *P* = 0.016) significantly increased recurrence risk.

Longer AF course (OR = 1.014, *P* = 0.005) and larger pre-operative LAAPD (OR = 1.108, *P* = 0.001) were positively correlated with recurrence, while lower albumin was also a risk factor (OR = 0.942, *P* = 0.048). Other variables-including sex, age, smoking, drinking, hypertension, diabetes, previous stroke, intra-operative rhythm conversion methods, number of electrical cardioversions, and most biochemical indices-were not significantly associated with recurrence (all *P* > 0.05).

Multivariate LR identified coronary artery disease (OR = 2.276, *P* = 0.043), heart failure (OR = 2.842, *P* = 0.044), pre-operative severe valvular regurgitation (OR = 4.831, *P* = 0.001), intra-operative myocardial injury (OR = 27.252, *P* < 0.001), longer AF course (OR = 1.024, *P* = 0.002), and increased pre-operative LAAPD (OR = 1.108, *P* = 0.018) as independent risk factors for post-operative AF recurrence. In contrast, additional ablation strategies (OR = 0.075, *P* = 0.019) and left atrial appendage occlusion (OR = 0.095, *P* < 0.001) were associated with significantly reduced recurrence risk, suggesting that comprehensive ablation and LAA management may help maintain sinus rhythm. Other variables, such as mitral isthmus ablation, cryoablation, and albumin levels, were not independently associated with recurrence in this model (all *P* > 0.05, Supplementary Table S7).

### Model performance evaluation

**Figure 3:**
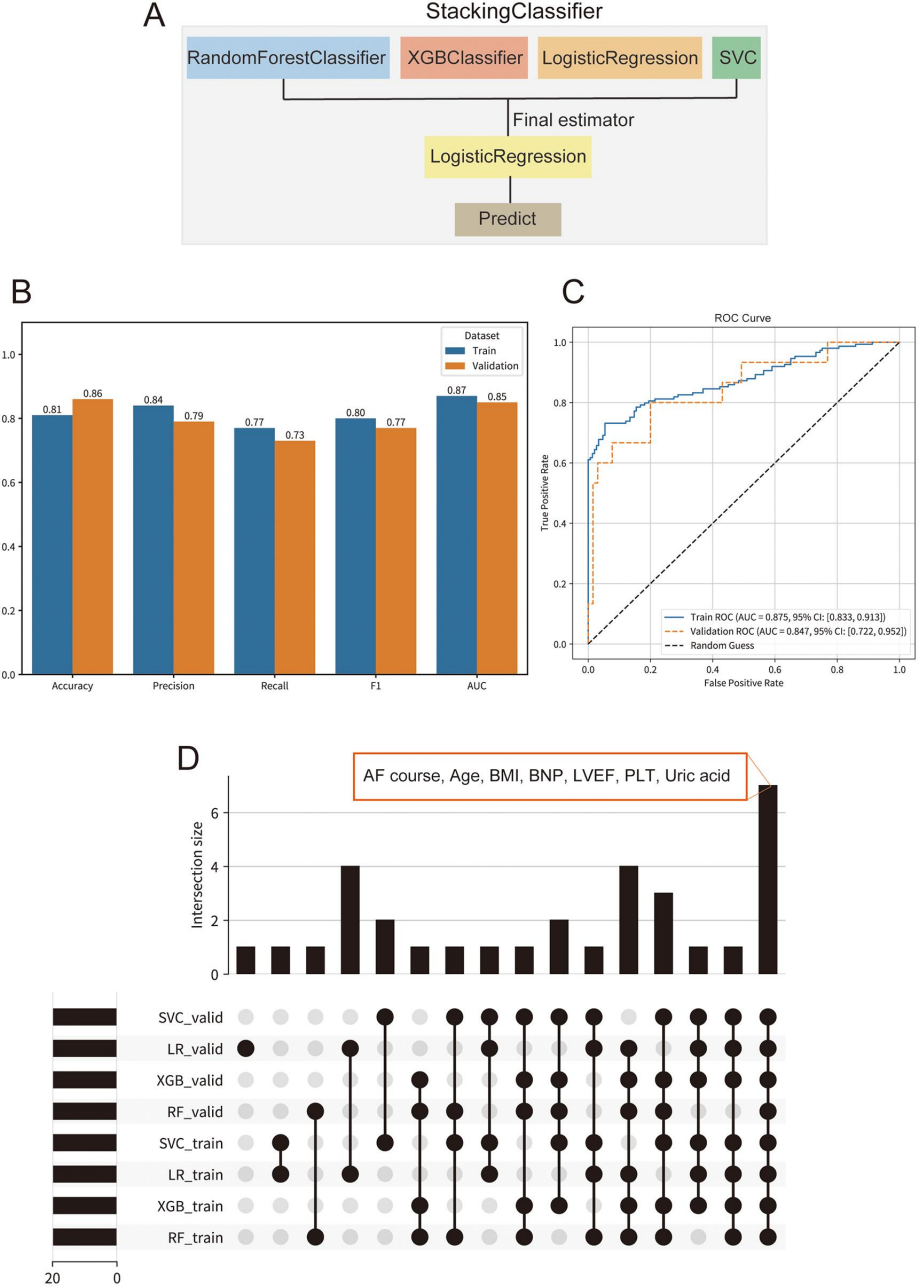
Performance and interpretability of the stacking ensemble model for predicting atrial fibrillation (AF) recurrence. **(A)** Schematic of the stacking ensemble model architecture, comprising random forest classifier (RF), XGBoost classifier, logistic regression (LR), and **support vector machine** (SVM) as base learners, with LR as the final estimator. **(B)** Comparison of key evaluation metrics, including accuracy, precision, recall, F1-score, and area under the curve (AUC). **(C)** Receiver operating characteristic (ROC) curves for the stacking model on training and validation sets. **(D)** UpSet plot illustrating the intersection of the top 20 SHAP-derived important features across base learners (RF, XGB, LR, SVM) in both training and validation sets.

After GA-based hyperparameter optimization, a Stacking ensemble model consisting of RF, XGBoost, LR, and SVM was constructed (Figure 3A). Model performance was assessed on both the training and validation sets using accuracy, precision, recall, F1 score, and ROC-AUC (Figure 3B). To assess the model's discriminative ability, ROC curves for the Stacking model were plotted for both the training and validation sets (Figure 3C). The AUC was 0.875 (95% CI: 0.833–0.913) for the training set and 0.847 (95% CI: 0.722–0.952) for the validation set, indicating excellent discrimination. The confusion matrix and decision curve analysis (DCA) on the validation set demonstrated strong performance of the model (Supplementary Figures S1A,B).

### Feature importance and interpretation of the stacking model's base learners

To enhance interpretability, SHAP analysis was conducted for each base learner (LR, RF, SVM, XGBoost) in the Stacking model across both training and validation sets. The results showed strong consistency in feature importance between datasets for all models, highlighting overall model stability (Supplementary Figures S2A–D, S3A–D). Key features identified across models included AF course, left atrial appendage occlusion, LVEF, age, weight, BMI, and PLT-all of which are well-supported by prior studies as risk factors for AF recurrence. Metabolic and functional markers such as uric acid, LDL-C, FBG, BNP, and creatinine, as well as procedural factors like additional ablation and mitral isthmus ablation, were also among the top predictors, suggesting that atrial remodeling, cardiac load, metabolic status, and ablation strategy collectively influence recurrence risk. These findings underscore the value of SHAP-based interpretation for identifying clinically meaningful predictors and characterizing high-risk patients for AF recurrence.

### Key consensus features identified by multi-model SHAP analysis

To enhance model stability and interpretability, intersection analysis of the top 20 SHAP features from the four base learners (XGB, RF, LR, SVM) in both training and validation sets was performed (Figure 3D). Seven features consistently emerged across models and datasets: AF course, age, BMI, BNP, LVEF, PLT, and uric acid. These consensus features are well-established risk factors for AF recurrence in the literature and have strong biological and clinical relevance.

## Discussion

This study demonstrates that adding EI-VOM and linear ablation to CPVI in patients with PeNVAF significantly reduces recurrence rates, improves quality of life, and enhances postoperative cardiac function, without affecting the intraoperative conversion rate. In PeNVAF, the substrate for abnormal electrical activity is more complex; therefore, targeting non-pulmonary vein triggers and abnormal substrates beyond standard CPVI can further support sinus rhythm (14). Additional ablation strategies, including routine use of EI-VOM, broaden the ablation area and improve efficacy. EI-VOM enhances PVI by targeting epicardial regions difficult to reach with conventional ablation, increases the durability of CPVI, and may reduce the need for further endocardial ablation (15–17). It also facilitates mitral isthmus block, addresses residual conduction due to anatomic variability, and provides rapid autonomic denervation (18). Studies have shown that EI-VOM leads to durable atrial lesions and improves outcomes by reducing conduction recovery and shortening ablation times (19). For patients with complex VOM anatomy or poor ablation response, other ablation strategies, such as endocardial ablation or intracoronary sinus ablation, can be combined to enhance the success rate of mitral isthmus block (20).

### Safety of EI-VOM combined with linear ablation

This study found no significant difference in intraoperative or postoperative complications between the two groups, indicating comparable safety for both ablation strategies. Previous research has shown that extensive ablation can impair left atrial function; however, ethanol infused via the VOM mainly affects targeted regions, expanding the ablation area without widespread damage. Kamakura et al. (15) found that EI-VOM-induced low-voltage zones were concentrated primarily at the left atrial ridge and mitral isthmus, involving less than 8% of the atrial surface in 90% of patients, suggesting focused lesion formation. Aranyo et al. (21) reported that while most patients developed new low-voltage areas and LGE-CMR scars after EI-VOM, there was no significant change in left atrial function parameters, supporting the safety of VOM ethanol ablation. Although EI-VOM can prolong overall procedure time, it may reduce the need for additional radiofrequency energy and does not increase the risk of procedural complications. In addition, radiofrequency power–delivery strategy may materially influence lesion geometry, transmurality, and long-term durability, particularly when ablation extends beyond pulmonary vein isolation (e.g., posterior wall ablation or broader substrate modification). Differences in power/time settings and lesion indexing can affect lesion continuity and the likelihood of reconnection, thereby modulating rhythm outcomes. We therefore interpret our findings in the context that procedural efficacy may reflect not only lesion set selection but also the RF power strategy used to achieve durable lesions (22).

Notably, there were no cases of pericardial tamponade requiring intervention in this study, likely due to optimized perioperative anticoagulation, precise intraoperative technique, and careful postoperative management. This aligns with expert consensus and meta-analyses indicating that, in experienced centers, serious complications are rare even with complex ablation strategies (23).

### Risk factors for AF recurrence

Numerous studies have confirmed various risk factors for AF recurrence, including AF history, laboratory and imaging findings, cardiac structural abnormalities, and comorbidities—findings consistent with this study. Machine learning models often identify variables such as age, anthropometric measures, blood pressure, comorbidities, and specific cardiac structural parameters (e.g., atrial enlargement, fibrosis, and valvular regurgitation) as predictors of recurrence.

First, both univariate and multivariate analyses in this study found that increased LAAPD and significant valvular regurgitation were associated with higher AF recurrence risk. LAAPD is a key marker of atrial size and is linked to adverse cardiovascular outcomes (24). AF can lead to atrial enlargement and functional mitral or tricuspid regurgitation, which in turn exacerbates atrial overload and remodeling (25, 26). Our six-month echocardiography follow-up showed reductions in LA diameter and improvement in regurgitation severity after ablation, suggesting reverse cardiac remodeling. This aligns with previous studies showing that restoring sinus rhythm after ablation can reverse atrial enlargement and decrease valve regurgitation, sometimes obviating the need for surgery in patients with severe functional valve disease (27, 28).

Second, postoperative management also plays a critical role. Hyperuricemia promotes myocardial fibrosis and atrial remodeling, increasing AF risk (29). Studies have shown that serum uric acid levels decrease after AF ablation, especially in persistent AF, and that choice of uric acid–lowering therapy can impact AF recurrence risk (30). For example, febuxostat has been linked to a higher risk of AF in older adults compared to allopurinol, and certain antihypertensive agents may be preferable for patients at risk (31, 32).

Finally, the main risk of AF is thromboembolism, with 90% of thrombi in NVAF patients originating from the LAA (33). Reduced LAA emptying velocity further increases thrombus risk. Catheter ablation combined with left atrial appendage closure (LAAC) has been proven safe and effective, and ablation itself improves left atrial structure and function (34, 35). The impact of LAAC on atrial function remains debated (36–39), but our study confirms that combined ablation and LAAC lead to improved left atrial function postoperatively.

### Development of a ML model for predicting AF recurrence

AF recurrence after PeAF ablation is a complex, multifactorial issue involving baseline characteristics, atrial substrate, procedural strategies, and postoperative management. In recent years, ML techniques have provided new tools to enhance prediction accuracy and enable individualized therapy, though studies focusing on AF recurrence prediction remain limited. Research comparing different ablation strategies for PeAF is also scarce. One international study (40) demonstrated excellent performance of ML models, with AUCs of 0.935, 0.855, and 0.965 for low-, medium-, and high-risk groups, respectively, effectively identifying patients at high risk for progression to permanent AF after ablation. Previous echocardiography-based studies have found associations between parameters such as left atrial volume, diastolic dysfunction, ventricular wall thickness, and total atrial conduction time with incident AF. The ML model developed in this study provides a means to predict recurrence after PeAF ablation, enabling identification of high-risk patients for tailored ablation strategies and intensified postoperative management. We developed a preliminary stacking-ensemble ML model to predict six-month recurrence after PeAF ablation. The model's most influential features (via SHAP analysis) were largely established clinical factors (e.g., age, AF duration, BMI, BNP, LVEF, atrial size). While this confirms known predictors, our model provides a framework for individualized risk stratification in this specific patient group (41). High-risk patients (as identified by the model) could be targeted for more aggressive ablation strategies (e.g., adjunctive VOM ethanol, additional lines) and closer follow-up. However, we emphasize that these findings are exploratory: without external validation, the model's performance may be optimistic, and its added clinical utility beyond existing risk scores remains to be proven.

### Study limitations and future directions

Several limitations temper our conclusions. First, the retrospective, single-center design inherently limits causal inference. We used PSM to balance measured covariates between groups, but PSM only adjusts for observed factors (42). Unmeasured confounders, such as operator preference or subtle anatomical differences, may still influence outcomes. In particular, variation in ablation energy sources (RF vs. cryoablation) and the composition of adjunctive lesions (VOM ethanol, lines, vein ablations, etc.) was not fully controlled. We combined these into a pragmatic “CPVI + PLUS” strategy, but our sample size precluded reliable subgroup analysis of each component's independent effect. As such, we refrain from attributing benefit to any single element; future larger studies will be needed to disentangle the contributions of VOM ethanol, linear lines, CS/SVC ablation, and LAAC. Second, the follow-up period was relatively short (six months). In persistent AF, late recurrences beyond six months are common, so our outcome reflects only early success. We acknowledge that longer-term efficacy remains to be seen. We are continuing to accrue cases and plan extended follow-up to assess durability. Third, our rhythm monitoring was limited to routine ECGs and symptom-prompted assessments. Intermittent monitoring can miss asymptomatic AF episodes: for example, studies using implantable monitors have shown that standard 3-month ECGs capture only about one-third of AF recurrences (43). Thus, our reported success rates may overestimate true freedom from AF. More intensive monitoring (e.g., routine Holters or continuous implants) in future work would improve outcome ascertainment. Finally, the ML component warrants caution. Using SMOTE oversampling, extensive hyperparameter tuning, and a multi-model stacking ensemble on a modest dataset heightens overfitting risk. Indeed, synthetic oversampling can create misleading patterns that do not generalize (44). Our model's performance, while internally validated, must be confirmed in external cohorts. We are collaborating with other centers to collect multicenter data for rigorous external validation and calibration. Until then, the model should be viewed as a preliminary, hypothesis-generating tool. In summary, CPVI + PLUS ablation for PeNVAF showed promise in reducing short-term recurrence and improving atrial reverse remodeling, without added procedural risk. These observational findings support a strategy of more extensive substrate targeting in selected patients, but they require confirmation in larger, prospective studies. Future research will refine which adjuncts (VOM ethanol, linear lines, LAA closure, etc.) are most beneficial, and will integrate improved rhythm monitoring and predictive modeling to optimize patient-specific ablation strategies.

## Conclusion

CPVI + PLUS (EI-VOM plus linear ablation) was associated with better 6-month arrhythmia-free survival, cardiac function, and quality of life, without an observed increase in intraoperative complications. Multivariable analyses suggested CPVI + PLUS was linked to lower recurrence risk, whereas heart failure, significant valvular regurgitation, and longer AF duration were linked to higher risk. The stacking model showed promising performance in internal validation, but given the retrospective single-center design, short follow-up, and lack of external validation, it should be considered exploratory and not ready to support **“**precise” risk stratification or individualized therapy.

## Data Availability

The original contributions presented in the study are included in the article/Supplementary Material, further inquiries can be directed to the corresponding author.
