# Supplementary figures and images for: A retrospective cohort and machine learning study on the efficacy, safety, and recurrence prediction of combined EI-VOM and linear ablation in persistent Non-valvular atrial fibrillation
Source: Front Cardiovasc Med. 2026 Feb 2;13:1689942. doi: 10.3389/fcvm.2026.1689942 (PMC12908034; doi:10.3389/fcvm.2026.1689942)

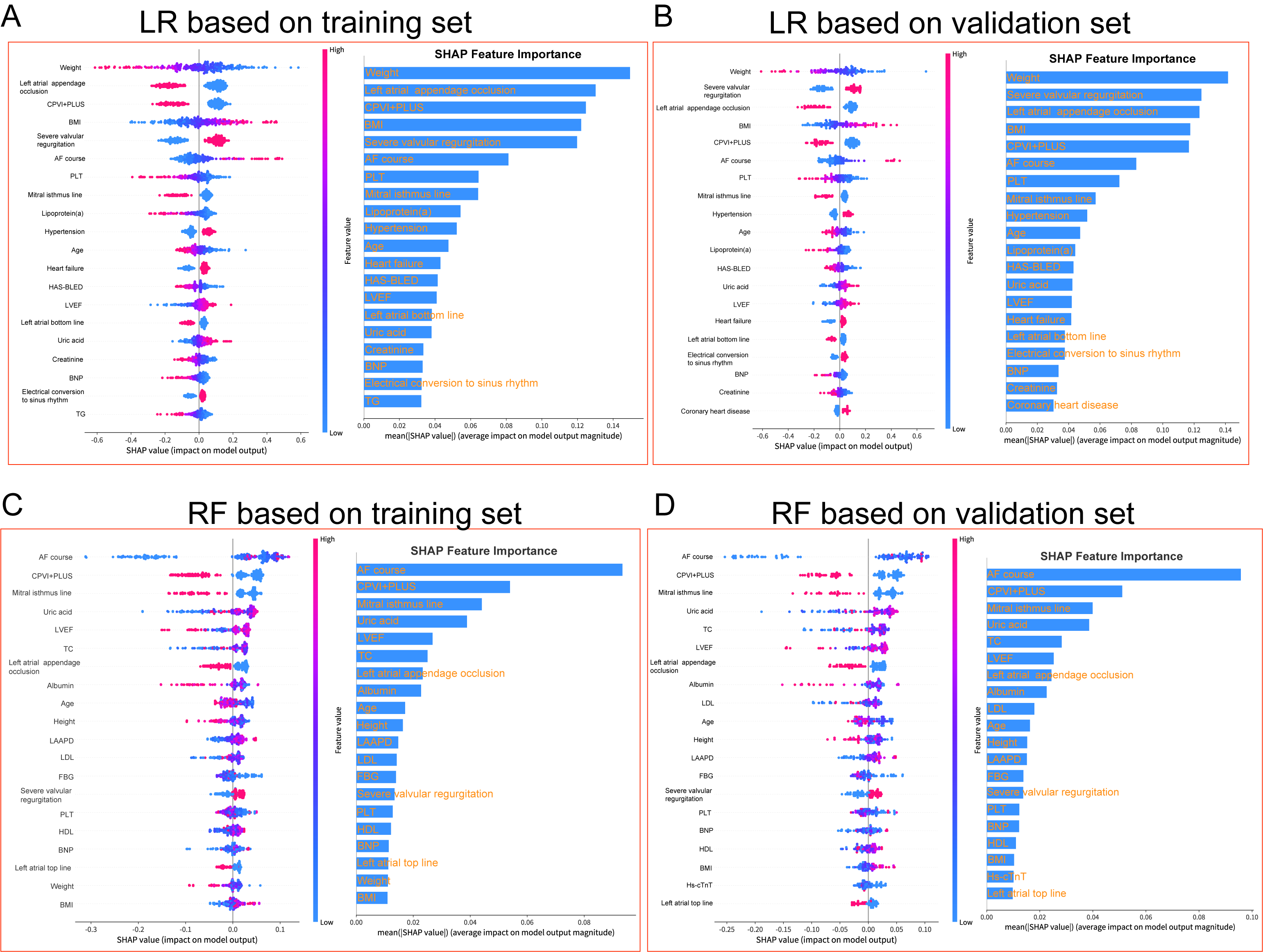

Supplement: Supplementary file 2 [file Image1.tif]

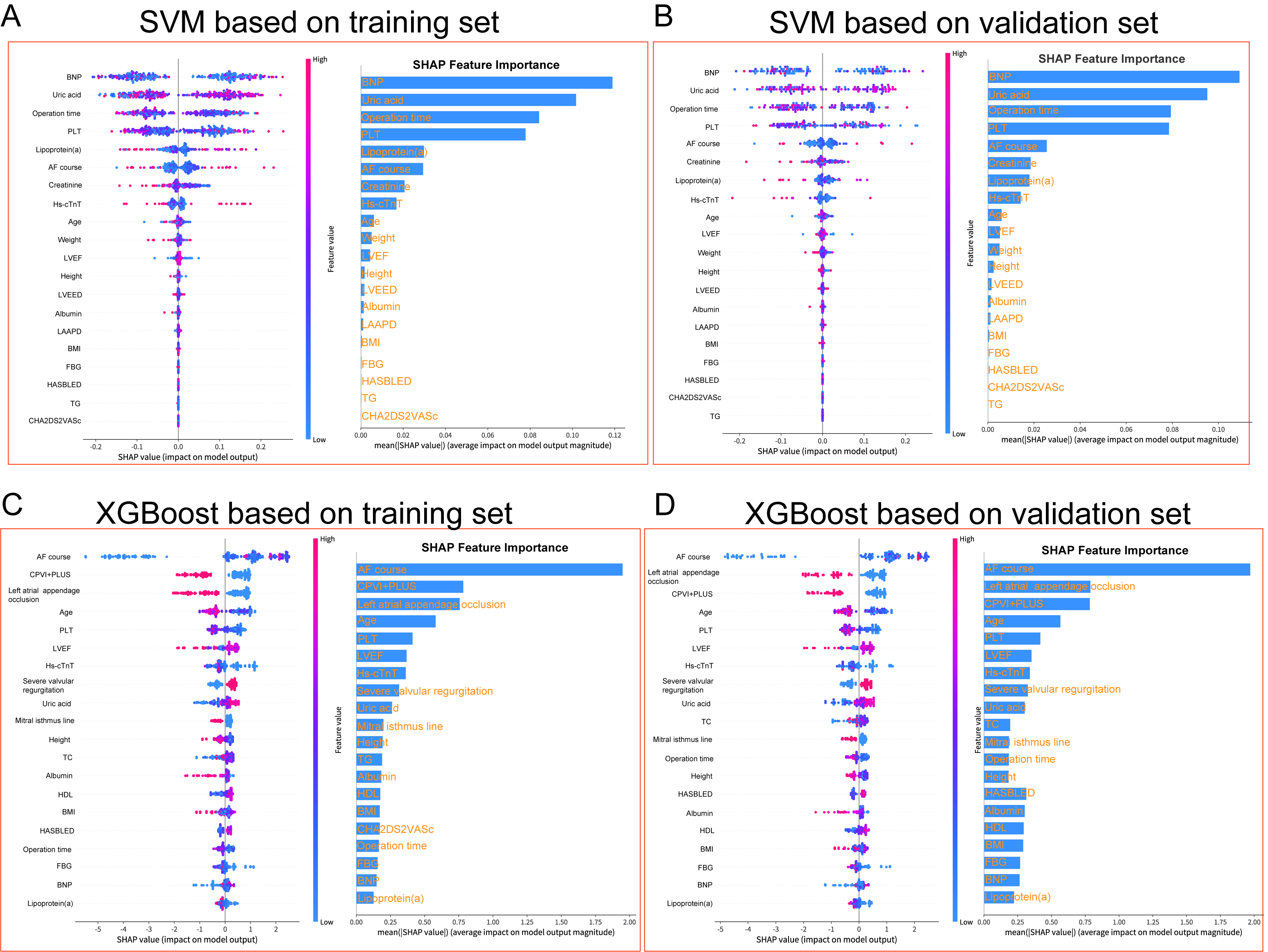

Supplement: Supplementary file 3 [file Image2.tif]

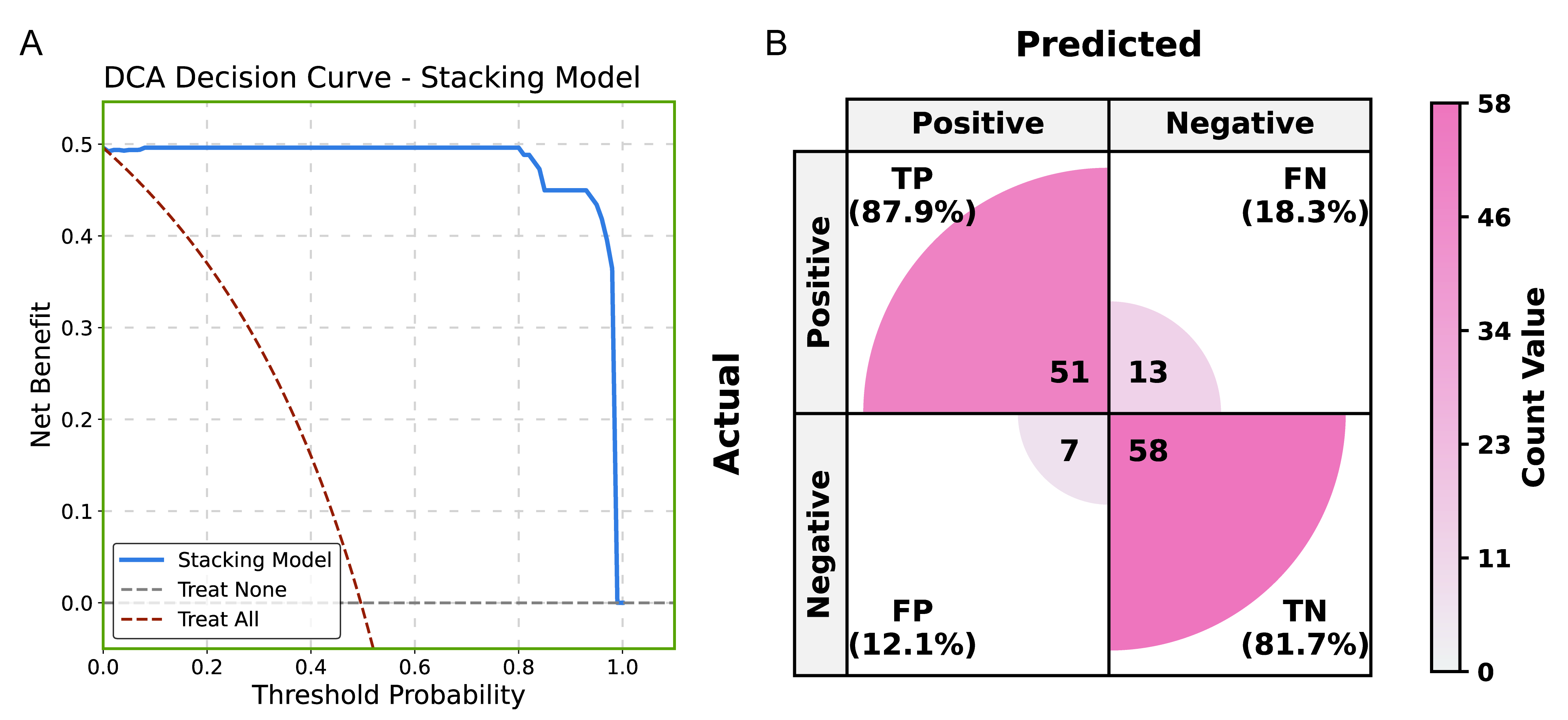

Supplement: Supplementary file 4 [file Image3.tif]
